# Failure to Achieve 70% of Recommended Protein Intake at One Year Predicts 13-Fold Higher Mortality After Gastrectomy

**DOI:** 10.3390/nu18010120

**Published:** 2025-12-30

**Authors:** Jou-Huai Lin, Shao-Ciao Luo, Li-Chun Liu, Ya-Ling Wang, Chiann-Yi Hsu, Pin-Kuei Fu

**Affiliations:** 1 Department of Food and Nutrition, Taichung Veterans General Hospital, Taichung City 40705, Taiwan; 2 Division of General Surgery, Department of Surgery, Taichung Veterans General Hospital, Taichung City 40705, Taiwan; 3 Biostatistics Task Force, Department of Medical Research, Taichung Veterans General Hospital, Taichung City 40705, Taiwan; 4 Division of Clinical Research, Department of Medical Research, Taichung Veterans General Hospital, Taichung 40705, Taiwan

**Keywords:** gastric cancer, protein intake, post-gastrectomy nutrition, gastrectomy, survival

## Abstract

**Background and Aims:** Gastric cancer remains a major health burden in East Asia. Gastrectomy is a primary treatment, yet postoperative malnutrition—particularly inadequate protein intake—adversely affects outcomes. This study assessed the association between achieving ≥70% of the recommended protein intake one year after gastrectomy and three-year survival. **Methods:** In this prospective, single-center, observational study, 69 patients with newly diagnosed gastric cancer who underwent gastrectomy between January 2021 and August 2023 were enrolled. Four patients who died within one year postoperatively were excluded, leaving 65 patients for analysis. Protein intake achievement rate (PIAR) at 12 months was calculated based on a recommended intake of 1.2 g/kg/day, and patients were stratified as PIAR ≥ 70% or <70%. Overall survival was analyzed using time-to-event methods, with a median follow-up of 2.1 years. **Results:** Among the 65 patients (median age 62 years, IQR 56–68; 56.9% male), 75.4% underwent subtotal gastrectomy. At 12 months, 7 patients (10.8%) failed to achieve a PIAR ≥ 70%. Compared with patients achieving adequate protein intake, those with inadequate intake more frequently underwent total gastrectomy (71.4% vs. 19.0%, *p* = 0.008) and had advanced-stage disease (Stage III–IV: 85.7% vs. 39.7%, *p* = 0.039). Kaplan–Meier analysis demonstrated significantly lower survival in the inadequate protein group, with a hazard ratio of 13.02 (95% CI 2.53–66.93); the wide confidence interval reflects the small number of patients with inadequate intake (n = 7). **Conclusions:** Failure to achieve ≥70% of recommended protein intake one year after gastrectomy is a strong independent predictor of mortality, associated with a 13-fold higher risk of death. Nutritional monitoring and early intervention are crucial, particularly for patients with total gastrectomy or advanced disease.

## 1. Introduction

Gastric cancer is the fifth most common malignancy worldwide and the third leading cause of cancer-related mortality, with East Asian populations bearing a disproportionately high burden [1,2]. Despite advances in multimodal therapies, gastrectomy remains the cornerstone of curative treatment for localized and locally advanced disease [3,4].

The post-gastrectomy period is characterized by profound nutritional challenges resulting from anatomical and physiological alterations, including loss of gastric reservoir function, disruption of neurohumoral regulation, and changes in gastrointestinal continuity [5,6]. These alterations commonly lead to reduced oral intake, altered dietary patterns, and impaired nutrient handling. Among these sequelae, loss of lean body mass (LBM) and inadequate protein intake have emerged as clinically relevant factors associated with delayed recovery, reduced tolerance to adjuvant therapy, and adverse clinical outcomes. Protein intake plays a central role in postoperative recovery by supporting wound healing, immune function, and preservation of skeletal muscle mass. Previous studies have shown that early postoperative loss of skeletal muscle mass or LBM is associated with poorer prognosis in patients with gastric cancer. In particular, insufficient protein intake during the early postoperative period has been linked to accelerated muscle loss and compromised continuation of adjuvant chemotherapy. Recent evidence further suggests that not only total protein intake but also intake distribution may influence outcomes, as consumption of at least one-third of daily protein requirements at breakfast during the early postoperative phase attenuates LBM loss after distal gastrectomy [7,8,9]. Collectively, these changes may culminate in protein–energy malnutrition, which is strongly associated with unfavorable clinical outcomes [10,11].

Current international guidelines recommend an increased protein intake—generally at least 1.2 g/kg/day—for patients undergoing major gastrointestinal surgery or cancer treatment, to counteract catabolism and preserve functional status [12,13,14]. However, achieving these targets remains challenging in real-world settings, particularly during long-term postoperative follow-up. Although the adverse impact of malnutrition on cancer outcomes is well recognized, evidence linking specific protein intake thresholds to long-term survival after gastrectomy remains limited.

In nutritional research, the concept of achieving a minimum proportion of estimated nutritional requirements has been widely used to distinguish clinically meaningful adequacy from insufficiency. While major guidelines such as ESPEN [15] do not mandate a specific percentage of protein intake for postoperative cancer patients, a threshold of approximately 70% of recommended or estimated requirements has been adopted in observational studies as a pragmatic benchmark. This threshold reflects a level below which sustained nutritional deficits, persistent muscle catabolism, and impaired recovery have been observed in high-risk populations, without assuming that full (100%) target intake is consistently attainable. Importantly, this benchmark represents clinical significance rather than a guideline-mandated target, and its relevance in the context of long-term postoperative recovery after gastrectomy has not been systematically evaluated.

Most previous studies in gastric cancer populations have focused on weight loss or composite nutritional indices, rather than macronutrient-specific intake patterns [16,17]. Furthermore, many investigations relied on cross-sectional assessments or short-term follow-up, limiting insights into the sustained impact of nutritional adequacy on survival. Identifying clinically relevant, long-term nutritional markers may therefore help inform proactive nutritional strategies and improve survivorship care.

This prospective cohort study examined the association between achieving ≥70% of recommended protein intake at one year after gastrectomy and overall survival in patients with gastric cancer. We hypothesized that failure to reach this threshold would be associated with poorer long-term survival, particularly among clinically high-risk subgroups, while acknowledging the multifactorial nature of postoperative outcomes.

## 2. Materials and Methods

### 2.1. Study Design and Population

This prospective observational cohort study was conducted at Taichung Veterans General Hospital, central Taiwan. The protocol was approved by the Institutional Review Board (IRB No. CE20384B), and all procedures adhered to the principles of the Declaration of Helsinki. Written informed consent was obtained from all participants prior to enrollment.

Between January 2021 and August 2023, consecutive patients with newly diagnosed gastric adenocarcinoma who were evaluated for surgical resection were screened. Inclusion criteria were: (1) histologically confirmed gastric adenocarcinoma; (2) clinical stage I–IV disease deemed resectable with curative intent; and (3) age ≥ 18 years. Exclusion criteria were: (1) unresectable disease or distant metastases; (2) prior gastric surgery; and (3) concurrent active malignancy.

### 2.2. Study Procedures and Follow-Up

**Figure 1:**
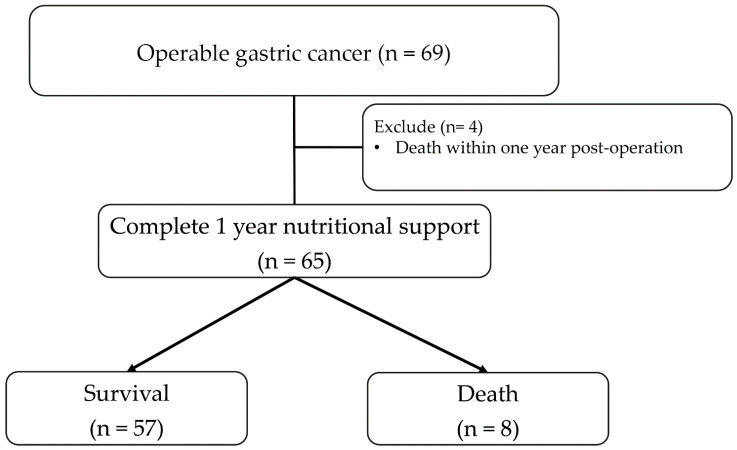
Flowchart of the registry study.

The study flowchart is shown in Figure 1. A total of 69 eligible patients underwent curative-intent gastrectomy. Four patients who died within 12 months postoperatively were excluded from the primary analysis to allow evaluation of one-year nutritional parameters, resulting in a final analytical cohort of 65 patients. Of these, 75.4% underwent subtotal gastrectomy and 24.6% total gastrectomy. Follow-up assessments were scheduled at 3, 6, and 12 months postoperatively, and every 3–6 months thereafter, with survival status monitored through 36 months.

### 2.3. Nutritional Assessment

Nutritional status was assessed at baseline (preoperatively) and at 12 months postoperatively. Protein intake achievement rate (PIAR) was calculated as:

PIAR = (actual protein intake/recommended protein requirement) × 100%.

The recommended protein requirement was defined as 1.2 g/kg/day using actual body weight. A PIAR of 70% was selected as the threshold for adequate protein intake based on clinical significance. Body mass index (BMI) was recorded at each assessment.

### 2.4. Statistical Analysis

Continuous variables were expressed as median with interquartile range (IQR), given non-normal distributions confirmed by the Shapiro–Wilk test. Categorical variables were summarized as frequencies and percentages. Between-group comparisons were performed using the Mann–Whitney U test for continuous variables and the chi-square or Fisher’s exact test for categorical variables, as appropriate.

The primary outcome was overall survival, analyzed using Kaplan–Meier methods with log-rank testing. Hazard ratios (HR) and 95% confidence intervals (CI) were estimated using Cox proportional hazards regression. Due to the limited number of events (n = 8 deaths), multivariable adjustment was not performed to avoid model overfitting; however, stratified analyses by age and sex were conducted. All statistical tests were two-sided, with significance defined as *p* < 0.05. Analyses were conducted using SPSS Statistics (version 22.0; IBM Corp., Armonk, NY, USA).

Sensitivity analyses were conducted to evaluate the robustness of the association between inadequate PIAR and 12-month mortality. Because the number of deaths was small, we performed an additional logistic regression analysis treating 12-month mortality as a binary outcome. This approach provides a model that does not rely on proportional hazards assumptions and yields a complementary effect estimate (odds ratio) that can be compared with the hazard ratio from the Cox model.

## 3. Results

### 3.1. Baseline Characteristics

Baseline characteristics and nutritional parameters of the 65 patients included in the analytical cohort are summarized in Table 1. The median age was 62 years (IQR: 56–68), with a slight male predominance (56.9%). Subtotal gastrectomy was performed in 75.4% of patients, and 24.6% underwent total gastrectomy. Disease stage was relatively balanced, with 55.4% having early-stage disease (Stage I–II) and 44.6% advanced disease (Stage III–IV).

### 3.2. Nutritional Changes from Baseline to One Year

Postoperative nutritional parameters demonstrated complex changes over the first year. Absolute energy and protein intake per kilogram of body weight increased modestly (energy: 25.4 → 28.7 kcal/kg; protein: 1.0 → 1.1 g/kg). However, this partially reflected significant weight loss, as BMI declined from 24.8 to 22.2 kg/m^2^ (*p* < 0.01). The proportion of patients achieving ≥70% of the recommended protein intake rose from 83.1% preoperatively to 89.2% at 12 months, indicating successful nutritional interventions in most patients.

### 3.3. Survival Outcomes

During a median follow-up of 2.1 years (IQR: 1.6–3.2), 8 patients (12.3%) died. Table 2 compares survivors and non-survivors. Although statistical significance was not reached due to limited power, non-survivors tended to be older (median 66 vs. 61 years, *p* = 0.089) and more frequently had advanced-stage disease (75.0% vs. 40.4%, *p* = 0.125).

### 3.4. Factors Associated with Inadequate Protein Intake

Table 3 compares patients stratified by protein intake adequacy at 12 months. Those with inadequate protein intake (PIAR < 70%, n = 7) exhibited strikingly different clinical features compared with patients achieving adequate intake (PIAR ≥ 70%, n = 58). Specifically, they had significantly higher rates of total gastrectomy (71.4% vs. 19.0%, *p* = 0.008) and advanced-stage disease (85.7% vs. 39.7%, *p* = 0.039), identifying clear high-risk subgroups requiring intensified nutritional support.

### 3.5. Impact of Protein Intake on Survival

**Figure 2:**
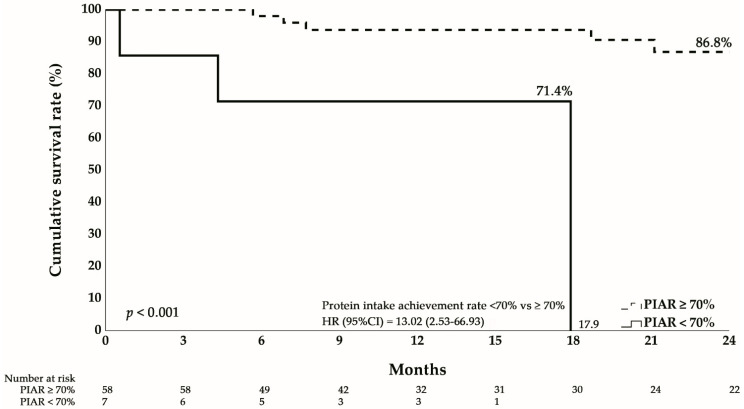
Kaplan–Meier survival curves depicting survival in gastric cancer patients, stratified by intake achievement rate of protein at 1 year after surgical intervention.

Kaplan–Meier curves (Figure 2) demonstrated markedly inferior survival in the inadequate protein group, with 24-month cumulative survival of only 17.9% compared to 86.8% in the adequate protein group (log-rank *p* < 0.001). Cox regression analysis confirmed this association, with inadequate protein intake conferring a hazard ratio of 13.02 (95% CI: 2.53–66.93, *p* = 0.002) for mortality.

Stratified analyses supported the robustness of this effect. The association remained significant among both females (HR = 16.12, 95% CI: 1.40–186.30, *p* = 0.026) and males (HR = 11.60, 95% CI: 1.03–131.18, *p* = 0.048), as well as in patients < 65 years (HR = 13.01, 95% CI: 1.75–96.85, *p* = 0.012). Although not statistically significant in patients ≥ 65 years, the effect size remained substantial (HR = 10.68, 95% CI: 0.66–171.51, *p* = 0.095), with limited power due to small sample size.

The results of the logistic regression sensitivity analysis were consistent with the primary Cox model. Inadequate PIAR remained significantly associated with 12-month mortality (OR = 7.95; 95% CI: 1.37–46.00), supporting the robustness of the association despite the limited number of events. Although the magnitude of the odds ratio differed from the hazard ratio, this discrepancy is expected due to the distinct statistical interpretations of the two effect measures and the low event count. Importantly, both analyses demonstrated the same direction of effect and statistical significance.

## 4. Discussion

This prospective cohort study demonstrates that failure to achieve ≥70% of the recommended protein intake at one year after gastrectomy was associated with poorer overall survival in patients with gastric cancer. While the magnitude of the observed association was substantial, the wide confidence interval reflects the limited number of events and underscores the need for cautious interpretation. Rather than implying causality, our findings highlight long-term protein intake adequacy as a clinically relevant marker of nutritional vulnerability during postoperative survivorship.

The clinical relevance of the 70% threshold warrants careful contextualization. Although optimal nutritional support aims to achieve 100% of recommended intake, real-world postoperative conditions often make this difficult to sustain [18,19]. The ≥70% benchmark represents a pragmatic and physiologically grounded threshold, distinguishing meaningful undernutrition from marginal shortfalls, rather than a guideline-mandated target. Similar intake-based thresholds have been used in observational nutrition studies to identify patients at risk of persistent catabolism and adverse outcomes [20,21,22,23]. Our findings suggest that sustained protein intake below this level at one year may identify patients who fail to recover nutritionally despite completion of acute surgical and oncologic treatments.

Our analysis also highlights a high-risk phenotype—patients undergoing total gastrectomy or presenting with advanced disease. The markedly higher prevalence of inadequate protein intake among total gastrectomy patients (71.4% vs. 19.0% with subtotal gastrectomy) reflects the profound nutritional consequences of complete gastric removal [24,25,26]. Similarly, the predominance of advanced-stage disease among the inadequate intake group likely reflects increased metabolic demands, treatment-related toxicities, and cancer-associated cachexia [27,28]. These subgroups may particularly benefit from intensive nutritional monitoring and preemptive supplementation.

The 12-month assessment point is clinically meaningful. By this time, patients have generally recovered from acute surgery, completed adjuvant therapy, and stabilized dietary patterns [29,30]. Our finding that protein adequacy at this time strongly predicts subsequent survival suggests that the one-year mark represents an optimal window for prognostic evaluation and intervention. Incorporating structured nutritional assessments at baseline and at 12 months could enhance clinical decision-making and facilitate timely interventions.

These results support a risk-stratified approach to nutritional management. High-risk patients (total gastrectomy, Stage III–IV disease) warrant intensive follow-up with a low threshold for nutritional supplementation. Standard-risk patients (subtotal gastrectomy, Stage I–II disease) should undergo regular monitoring, with intervention triggered by protein intake below 70%. Importantly, protein achievement rates provide a practical, individualized measure that accounts for body weight variations and can be integrated into routine follow-up.

Several limitations should be acknowledged. The small number of patients with inadequate intake (n = 7) and deaths (n = 8) limited statistical power and resulted in imprecise effect estimates, as reflected by wide confidence intervals. The single-center Taiwanese cohort may limit generalizability to other ethnic populations or healthcare systems with different dietary practices and postoperative care pathways. Although dietary intake at one year was assessed using structured, dietitian-administered recall, recall bias cannot be fully excluded. In addition, histological sub-classification according to WHO or Lauren criteria was not uniformly available, precluding subtype-specific analyses. While early postoperative mortality and major complications were not observed within 30 days in this cohort, later medical and oncologic events may still have influenced long-term outcomes. Finally, as an observational study, causal inference cannot be established, and residual confounding from unmeasured factors remains possible.

Despite these limitations, the association between inadequate protein intake and mortality remained directionally consistent across sensitivity analyses, including logistic regression approaches that do not rely on time-to-event assumptions. Although effect estimates differed in magnitude, as expected with small event counts, this consistency supports the robustness of the observed association while reinforcing the need for conservative interpretation.

In summary, failure to achieve ≥70% of recommended protein intake at one year after gastrectomy was associated with poorer survival in gastric cancer patients. This threshold may serve as a clinically meaningful indicator of long-term nutritional vulnerability, rather than a definitive prognostic determinant. Our findings underscore the importance of systematic nutritional assessment and sustained, individualized support during survivorship, particularly for patients undergoing total gastrectomy or presenting with advanced disease. Larger, multicenter studies with standardized nutritional interventions are warranted to validate these observations and to determine whether improving long-term protein intake can translate into better clinical outcomes.

## 5. Conclusions

Failure to achieve ≥70% of the recommended protein intake at 12 months after gastrectomy was associated with poorer overall survival in patients with gastric cancer. Although the estimated effect size was substantial, the wide confidence interval reflects the limited number of events and warrants cautious interpretation. These findings indicate that long-term nutritional adequacy may represent a clinically meaningful marker of persistent postoperative vulnerability, particularly among patients undergoing total gastrectomy or presenting with advanced-stage disease. Larger multicenter studies with standardized nutritional assessment and intervention protocols are required to validate the prognostic relevance of this threshold. From a clinical perspective, the results highlight the importance of ongoing, dietitian-led nutritional monitoring and support during long-term follow-up after gastrectomy.

## Figures and Tables

**Table 1:** Demographic characteristics of enrolled patients with gastric cancer.

	Total (n = 65)
Age, median (IQR)	62 (56–68)
Sex, n (%)	
Female	28 (43.1%)
Male	37 (56.9%)
BMI (kg/m^2^)	24.8 (21.6–27.8)
Gastrectomy, n (%)	
Total	16 (24.6%)
Subtotal	49 (75.4%)
Stage, n (%)	
Stage I&II	36 (55.4%)
Stage III&IV	29 (44.6%)
Preoperative, median (IQR)	
Energy intake/weight (kal/kg)	25.4 (22.2–28.8)
Protein intake/weight (g/kg)	1 (0.9–1.2)
Energy intake achievement rate (%)	95.9 (84.9–102.6)
Protein intake achievement rate (%)	89.2 (73.2–105)
Energy intake achievement rate < 70%, n (%)	7 (10.8%)
Protein intake achievement rate < 70%, n (%)	11 (16.9%)
Lab data (Preoperative), median (IQR)	
Alb (g/dL)	4 (3.7–4.3)
Hb (g/dL)	12.4 (10.3–14.3)
Fe (μg/dL)	54 (32–71)
TIBC (μg/dL)	328 (281–370)
Ferritin (ng/mL)	70.2 (21.9–158.7)
RBC folate (ng/mL)	927.7 (795.3–1113.9)
CRP (mg/dL)	0.1 (0.1–0.3)
B12 (pg/mL)	440 (340–863)
Death, n (%)	8 (12.3%)
Follow-up time (year), median (IQR)	2.1 (1.6–3.2)

**Table 2:** Comparison of baseline characteristics by survival status of enrollment.

	Alive (n = 57)	Death (n = 8)	*p* Value
Age, median (IQR)	61 (54–68)	66 (62–77)	0.089
Sex, n (%)			1.000
Female	25 (43.9%)	3 (37.5%)	
Male	32 (56.1%)	5 (62.5%)	
BMI (kg/m^2^)	22.8 (20.1–25.5)	21.2 (19.9–23.1)	0.299
Gastrectomy, n (%)			0.395
Total	13 (22.8%)	3 (37.5%)	
Subtotal	44 (77.2%)	5 (62.5%)	
Stage, n (%)			0.125
Stage I&II	34 (59.65%)	2 (25.0%)	
Stage III&IV	23 (40.35%)	6 (75.0%)	
Postoperative 12-month, median (IQR)			
Energy intake/weight (kal/kg)	28.9 (25.7–32.1)	26.2 (19.1–31.1)	0.263
Protein intake/weight (g/kg)	1.1 (1.0–1.3)	1.0 (0.8–1.2)	0.148
Energy intake achievement rate (%)	99.6 (90.3–108.3)	89.5 (69.4–103.9)	0.129
Protein intake achievement rate (%)	97.5 (86.3–105.1)	83.4 (65.3–101.0)	0.090
Energy intake achievement rate < 70%, n (%)	5 (8.8%)	2 (25.0%)	0.203
Protein intake achievement rate < 70%, n (%)	4 (7.0%)	3 (37.5%)	0.035 *
Lab data (Preoperative), median (IQR)			
Alb (g/dL)	4.1 (3.7–4.3)	3.7 (3.5–3.9)	0.041 *
Hb (g/dL)	12.6 (10.4–14.3)	10.4 (8.6–14)	0.212
Fe (μg/dL)	56 (34–76.5)	45 (29.3–68.8)	0.677
TIBC (μg/dL)	329 (278.5–370)	317 (283.5–371.8)	0.898
Ferritin (ng/mL)	71.8 (21.9–182.9)	55.9 (23.2–149.7)	0.665
RBC folate (ng/mL)	927.7 (792.4–1113.9)	957.6 (829.4–1339.3)	0.842
CRP (mg/dL)	0.1 (0.1–0.3)	0.1 (0.1–0.2)	0.825
B12 (pg/mL)	457 (346.8–923)	379.5 (327–497.5)	0.247

Mann–Whitney U test. Fisher’s exact test. * *p* < 0.05.

**Table 3:** Factors associated with intake achievement rate of protein 12 months after surgical intervention (post-op 12 months).

	Protein ≥ 70% (n = 58)	Protein < 70% (n = 7)	*p* Value
Age, median (IQR)	62 (56–68)	62 (47–71)	0.882
Sex, n (%)			0.224
Female	23 (39.7%)	5 (71.4%)	
Male	35 (60.3%)	2 (28.6%)	
BMI (kg/m^2^)	24.8 (21.5–27.3)	28.3 (23.9–31.7)	0.079
Gastrectomy, n (%)			0.00796
Total	11 (19%)	5 (71.4%)	
Subtotal	47 (81%)	2 (28.6%)	
Stage, n (%)			0.0388
Stage I&II	35 (60.3%)	1 (14.3%)	
Stage III&IV	23 (39.7%)	6 (85.7%)	
Protein intake/weight (g/kg)	1 (0.9–1.2)	1 (0.7–1.2)	0.397
Lab data (Preoperative), median (IQR)			
Alb (g/dL)	4 (3.7–4.2)	4 (3.6–4.3)	0.915
Hb (g/dL)	12.5 (10.4–14.3)	10.9 (9.5–14.6)	0.539
Fe (μg/dL)	53 (33–70)	62 (26–97)	0.790
TIBC (μg/dL)	328 (277–371)	330 (306–360.5)	0.649
Ferritin (ng/mL)	70.3 (24.5–191)	70.2 (17.5–122.7)	0.570
RBC folate (ng/mL)	910.4 (791.8–1098.6)	1106.2 (909.1–1288.7)	0.115
CRP (mg/dL)	0.1 (0.1–0.2)	0.2 (0.1–0.4)	0.077
B12 (pg/mL)	436 (333.5–849.5)	524 (362–954)	0.572

Mann–Whitney U test. Chi-Square test. Fisher’s exact test.

## Data Availability

The datasets used and analysed in the current study are available from the corresponding author on reasonable request due to privacy.
